# Supplementary material for: Index diagnoses of gastric intestinal metaplasia in the United States: patient characteristics, endoscopic findings, and clinical practice patterns at a large tertiary care center
Source: Ther Adv Gastroenterol. 2022 Sep 3;15:17562848221117640. doi: 10.1177/17562848221117640 (PMC9445457; doi:10.1177/17562848221117640)
Supplement: sj-docx-1-tag-10.1177_17562848221117640 – Supplemental material for Index diagnoses of gastric intestinal metaplasia in the United States: patient characteristics, endoscopic findings, and clinical practice patterns at a large tertiary care center [file sj-docx-1-tag-10.1177_17562848221117640.docx]

**Index diagnoses of gastric intestinal metaplasia in the United States: Patient characteristics, endoscopic findings, and clinical practice patterns at a large tertiary care center**

Sheeva K. Parbhu^1^, Shailja C. Shah^2,3^, Michael J. Sossenheimer^1^, John C. Fang^1^, Kathryn A. Peterson^1^, Andrew J. Gawron^1,4^

Author Affiliations

^1^Department of Internal Medicine, Division of Gastroenterology and Hepatology, University of Utah School of Medicine, Salt Lake City, Utah, USA

^2^Division of Gastroenterology, University of California San Diego, San Diego, CA

^3^Veterans Affairs San Diego Healthcare System, San Diego, CA

^4^Informatics, Decision-Enhancement, and Analytic Sciences Center, VA Salt Lake City Health Care System, Salt Lake City, Utah, USA

**Abbreviations**

AGA – American Gastroenterological Association

CPT – Current Procedural Terminology

EGD – Esophagogastroduodenoscopy

EMR – Electronic medical record

ESGE – European Society of Gastrointestinal Endoscopy

GIM – Gastric intestinal metaplasia

GIST – Gastrointestinal stromal tumor

HP – Helicobacter Pylori

ICD – International Classification of Diseases

MALT – mucosa-associated lymphoid tissue

NLP – Natural language processing

US – United States

**Correspondence**

Andrew J. Gawron

Division of Gastroenterology, Hepatology & Nutrition

30 North 1900 East, SOM 4R118

Salt Lake City, UT 84132

O: 801-581-5679

F: 801-581-7476

Email: andrew.gawron@hsc.utah.edu

**Abstract**

**Background:** Gastric intestinal metaplasia (GIM) is a premalignant gastric mucosal change that is often incidentally detected during esophagogastroduodenoscopy (EGD). Despite the established higher risk of gastric cancer associated with GIM, the incidence, prevalence, and outcomes data for GIM are limited in the United States (US), and practice patterns are highly variable.

**Objectives:** Our primary objectives were to accurately identify incident histology-confirmed GIM cases and determine patient characteristics, endoscopy findings, *Helicobacter pylori* (HP) detection and eradication treatment outcomes, as well as surveillance and follow-up recommendations.

**Design:** We conducted a retrospective cohort study using administrative data.

**Methods:** We first developed and validated a rule-based natural language processing (NLP) tool to identify patients with gastric intestinal metaplasia on gastrointestinal pathology reports between 2011-2016. We then performed a manual chart review of all EGD procedures and associated pathology notes to confirm cases and obtain clinically relevant data.

**Results:** 414 patients with an index diagnosis of GIM were confirmed (prevalence = 2.5% of patients undergoing any EGD). A majority (52.4%) of patients were non-Hispanic white. The most common indication for EGD was abdominal pain (46.9%). A majority (55%) did not receive specific follow-up recommendations or were asked to see their primary care provider. HP testing was documented in 86% of patients, and detected in 94 patients (prevalence = 26.4%). Treatment was documented in 94.7% of cases, and eradication confirmed in only 34.8% of these cases.

**Conclusion:** A large group of US patients with an index diagnosis of GIM was accurately identified. There was wide variability in clinical practice patterns including biopsy practice, HP treatment and eradication confirmation testing, and surveillance recommendations. This work demonstrates there is a major unmet need for quality improvement efforts to standardize care for patients with GIM, a premalignant condition, and inform future prospective studies in a US population.

**Keywords:** gastric intestinal metaplasia, gastric cancer, endoscopy, *Helicobacter pylori*, screening and surveillance

**Introduction**

Gastric cancer ranks as the 5^th^ most common cause of cancer and 4^th^ most common cause of cancer-related death worldwide, responsible for an estimated 1 million new cases and over 780,000 related deaths in 2019 alone, accounting for 8.2% of all cancer related deaths.[^1^](https://sciwheel.com/work/citation?ids=10431948&pre=&suf=&sa=0&dbf=0) In the United States (US), gastric cancer is the 2^nd^ most common luminal GI malignancy after colorectal cancer, with an estimated 26,380 new cases and 11,090 related deaths estimated to occur in 2022.[^2^](https://sciwheel.com/work/citation?ids=6437304&pre=&suf=&sa=0&dbf=0) Importantly, although the US is considered a low-incidence country overall, certain racial and ethnic minority groups have a much higher incidence.[^3,4^](https://sciwheel.com/work/citation?ids=2480997,9409962&pre=&pre=&suf=&suf=&sa=0,0&dbf=0&dbf=0) It was recently demonstrated that immigrants from high to low incidence countries retained their elevated risk of gastric cancer and related mortality.[^5^](https://sciwheel.com/work/citation?ids=7530995&pre=&suf=&sa=0&dbf=0) Indeed, the incidence of gastric cancer is at least 2-3 fold higher than US-born non-Hispanic Whites, with the incidence increasing in certain demographic groups.[^4,6,7^](https://sciwheel.com/work/citation?ids=9409962,4477347,4953358&pre=&pre=&pre=&suf=&suf=&suf=&sa=0,0,0&dbf=0&dbf=0&dbf=0)

Gastric intestinal metaplasia (GIM) develops in response to chronic gastric inflammation, with the most common trigger *Helicobacter pylori* (HP) infection. GIM is considered a premalignant condition with an estimated 0.16% baseline associated annual risk of gastric cancer, with certain modifying factors such as extent of GIM and persistent HP infection associated with an even higher risk of progression.[^8–10^](https://sciwheel.com/work/citation?ids=7944023,7944022,2280016&pre=&pre=&pre=&suf=&suf=&suf=&sa=0,0,0&dbf=0&dbf=0&dbf=0) Inflammation from HP infection is known to play a primary role in carcinogenesis, along with other factors including genetic (family history) and environmental (smoking, diet) factors.[^8^](https://sciwheel.com/work/citation?ids=7944023&pre=&suf=&sa=0&dbf=0) Because GIM can be reliably identified by gastroenterologists and pathologists, combined with the slow stepwise progression to dysplasia or cancer in a small minority of individuals, endoscopic surveillance of GIM offers a promising opportunity for early diagnosis of gastric cancer with markedly improved outcomes. Indeed, early gastric cancer prior to submucosal invasion is associated with greater than 95% 5-year survival compared to the dismal < 30% 5-year survival associated with more advanced disease. In countries with a high burden of gastric cancer, identification and surveillance of patients with premalignant lesions (ie GIM) can be a cost-effective way of decreasing the morbidity and mortality associated with the disease.[^11–13^](https://sciwheel.com/work/citation?ids=2806399,7531294,9418815&pre=&pre=&pre=&suf=&suf=&suf=&sa=0,0,0&dbf=0&dbf=0&dbf=0)

In the US, due to low overall prevalence of GIM, surveillance has not been shown to be effective in preventing cancer, although some subgroups of the population may benefit.^[13–15](https://sciwheel.com/work/citation?ids=9418815,9657736,5401558&pre=&pre=&pre=&suf=&suf=&suf=&sa=0,0,0&dbf=0&dbf=0&dbf=0)^ GIM is often detected incidentally from biopsies during esophagogastroduodenoscopy (EGD) performed for common symptoms. Incidence, prevalence and outcomes data are limited in the US due to an inability to accurately identify patients with GIM in the electronic medical records (EMR). Additionally, endoscopy and pathology reports are often separate and data acquisition can be challenging. Thus, there have been very few studies evaluating large cohorts of patients with GIM in the US.[^16^](https://sciwheel.com/work/citation?ids=1481682&pre=&suf=&sa=0&dbf=0) Two small surveys of US physicians have highlighted the variability of clinical practice patterns after diagnosis of GIM, and identified significant knowledge gaps in regards to ethnic populations who may be at increased risk for gastric cancer. [^17,18^](https://sciwheel.com/work/citation?ids=6992304,3195680&pre=&pre=&suf=&suf=&sa=0,0&dbf=0&dbf=0) A recent large study which evaluated risk factors in US Veterans identified older age, male sex, nonwhite race/ethnicity and current smoking status as non-endoscopic risk factors which were associated with GIM development.[^19^](https://sciwheel.com/work/citation?ids=13296233&pre=&suf=&sa=0&dbf=0)

Recently published professional society guidelines highlight the paucity of high-quality evidence guiding consistent recommendations surrounding GIM management, which is propagated further by difficulties in case ascertainment that would enable robust epidemiologic and outcomes studies.^[20,21](https://sciwheel.com/work/citation?ids=7699366,7969904&pre=&pre=&suf=&suf=&sa=0,0&dbf=0&dbf=0)^ The recent American Gastroenterological Association (AGA) guideline on GIM recommends against routine endoscopic surveillance of GIM due to lack of high-quality evidence, but acknowledges that certain demographic groups may benefit.[^20^](https://sciwheel.com/work/citation?ids=7699366&pre=&suf=&sa=0&dbf=0) There was stronger evidence to support treatment of HP in patients with GIM. Thus, the primary objectives of our study included creation of a valid and accurate method to identify patients with GIM diagnosed by EGD, to describe characteristics as well as EGD indications and findings of patients incidentally diagnosed with GIM, and to describe the clinical practice habits after diagnosis, including biopsy patterns and follow-up recommendations. Secondary objectives included assessing the prevalence of GIM in a cohort of patients undergoing EGD, defining the proportion of patients with GIM tested and treated for HP, and delineating the associated pathologic characteristics of GIM, based on a large academic tertiary care setting.

**Methods**

This retrospective cohort study was approved by the University of Utah Health Institutional Review Board. The reporting of this study conforms to the STROBE statement.[^22^](https://sciwheel.com/work/citation?ids=2432932&pre=&suf=&sa=0&dbf=0)

*Inclusion and exclusion*

We queried the institution’s data warehouse to identify all eligible patients. Eligible patients were those who underwent an EGD with current procedural terminology (CPT) codes (43200-43259) at the University of Utah (a tertiary referral center) between September 2011 and November 2016 and had gastric biopsies obtained during the procedure. Only patients with new (index cases) diagnoses of GIM were included. Individuals < 18 years old, individuals who underwent EGD but did not have gastric biopsies, individuals with evidence of prior GIM, and individuals with evidence of low- or high-grade dysplasia or gastrointestinal malignancy at the time of GIM diagnosis were excluded. The first diagnosis of GIM was considered the index diagnosis.

*Natural Language Processing*

A rules-based Pycon Text natural language processing (NLP) tool was applied to the entire document corpus of endoscopy and pathology notes to identify patients with a diagnosis of GIM.[^23^](https://sciwheel.com/work/citation?ids=5653968&pre=&suf=&sa=0&dbf=0) Natural language processing systems are a form of machine learning and are developed using a standard or “training” set of terms or phrases that provide the “dictionary” for the software to extract the information from text documents. In this case, phrases and terms from pathology results for intestinal metaplasia, including accounting for misspelling, were used to train the system. The software system was then validated iteratively to determine information extraction characteristics. The NLP system was developed using open source code and is available to use and reproduce at this link: <https://gitlab.chpc.utah.edu/endoqual/endoscopy-path-classify/-/tree/master>

*Data abstraction and case confirmation*

The electronic medical records (EMRs) of all patients meeting initial inclusion criteria, inclusive of clinical notes, endoscopic reports, and pathology reports, were manually reviewed in order to confirm the diagnosis of GIM and abstract additional details. Using a standardized data collection form, we recorded the following details at the time of endoscopic evaluation: age, sex, race/ethnicity (non-Hispanic white, non-Hispanic black, Hispanic, Asian, etc), smoking status (current, former, never), and family history of gastric cancer in a first-degree relative. Missing or unknown data were documented. The following endoscopic details were recorded: indication, gross findings, location of each biopsy site, including whether antrum/incisura and corpus biopsies were placed in separate containers. The following pathology details were recorded: locations of GIM (antrum, incisura, corpus/fundus, cardia), anatomic extent (corpus-extended GIM, defined as any GIM involving the corpus vs limited GIM, defined as GIM confined to the antrum/incisura[^8^](https://sciwheel.com/work/citation?ids=7944023&pre=&suf=&sa=0&dbf=0)), and any additional gastric pathology (e. g. dysplasia, cancer). We also manually abstracted other HP testing and eradication therapy details at any time following patients’ diagnosis of GIM; details included test modality, result, eradication therapy (yes/no) and eradication outcomes (eradicated successfully versus persistent infection). Follow-up recommendations after a confirmed index diagnosis of GIM were documented. No biopsies were re-reviewed for the purposes of this study.

Analyses and descriptive statistics were performed using STATA 10.0.

**Results**

A total of 16,505 distinct patients underwent a total of 23,404 procedures during the study time frame. The NLP tool initially identified 516 patients with a likely diagnosis of GIM, 510 (98.8%) of whom were confirmed to have histologic evidence of GIM on manual review of associated pathology reports. The F-measure, a surrogate for specificity for information extraction, of the NLP tool was 92%. An additional 20 patients (3.9%) had dysplasia or evidence of malignancy at the time of diagnosis and were excluded. After additional exclusion criteria were applied (**Figure 1**), a total of 414 patients with an index diagnosis of GIM were included for analysis.

The overall prevalence of incidentally diagnosed GIM in all patients undergoing endoscopy during the study time period was 2.5%. Baseline characteristics of the patients diagnosed with GIM and no neoplasia (N=414) are included in Table 1, along with comparative demographics of those diagnosed with concomitant neoplasia (N=20). The mean age was 59.3 years (range, 18-91 years), and the majority of patients were female (60.1%). With respect to race/ethnicity, most were non-Hispanic white (52.4%), followed by Hispanic (26.1%), Asian (9.7%) and Native American/Pacific Islander (5.6%), respectively. A majority were categorized as never-smokers.

The most common indication for EGD was abdominal pain/dyspepsia (46.9%), followed by gastro-esophageal reflux disease (22.2%), dysphagia (16.9%), iron deficiency anemia (8.5%) and nausea/vomiting (7.7%) **(Table 2**). The most common endoscopic finding among patients with GIM was gastritis (69.8%), with a minority reporting ulcerations/erosions (13.5%) or normal gastric mucosa (13%). The most common specified location of gastric biopsies were antrum and corpus combined in the same container (47.6%), followed by antrum alone (23.2%), fundus alone (12.8%) and corpus alone (2.9%). Nearly 25% had an unspecified location for gastric biopsies.

**Table 3** details the anatomic locations of confirmed GIM, as well as anatomic extent. An unspecified gastric location for GIM was observed in 43% of cases (e.g. “random gastric” biopsies), followed by antrum alone (34.1%), antrum and body combined (10.9%), and body alone (8.7%).

Follow-up recommendations varied widely (**Figure 2**). A majority of patients (55%) did not receive specific follow-up recommendations or were simply asked to follow-up with their primary care provider as opposed to their GI provider, while 38% of patients were offered repeat EGD (23% being recommended within 1 year and the remaining 15% recommended an EGD at an interval greater than 1 year). A minority (7%) of patients were offered a gastroenterology clinic visit to discuss these findings and decide upon a future care plan. Of the 97 patients who were recommended to have a repeat EGD within 1 year, 51% had this exam completed at our institution within that time frame.

A majority of patients (86%) did have HP testing based on immunohistochemical testing of pathology specimens. Of these, HP was detected in 94 patients (**Table 4**), with a prevalence of 26.4% in patients with an index diagnosis of GIM. Treatment was documented in 89 of these patients (94.7%), but unfortunately, most patients did not have eradication confirmation testing. Eradication was confirmed based on repeat testing in 31 patients (34.8%). The most commonly used test to confirm eradication was repeat biopsy (16/31 patients, 51.6%), followed by stool antigen test (8/31 patients, 25.8%) and breath test (7/31 patients, 22.6%).

In total, 20 patients were excluded because of findings of malignancy or dysplastic changes at the time of index diagnosis of GIM. The total malignancies (12) were as follows: adenocarcinoma (4), neuroendocrine tumor (4), mucosa-associated lymphoid tissue (MALT) lymphoma (2), gastrointestinal stromal tumor (GIST) (1), and metastatic hepatocellular carcinoma (1). The remaining 8 patients with high-grade dysplasia were all recommended to have a repeat EGD within one year, and 5 of these patients had documented follow-up within our EMR.

**Discussion**

In this large retrospective observational cross-sectional study, we identified marked practice pattern variability and heterogeneity in the management of index diagnoses of GIM. Despite GIM being a well-established premalignant condition, with an estimated baseline 0.16% annual risk of gastric cancer, the vast majority of patients diagnosed with GIM were not recommended for any GI follow-up nor further risk stratification to better determine their need for ongoing surveillance. Additionally, we successfully developed and validated an NLP tool among patients referred for routine EGD, and confirmed the diagnostic accuracy for incident GIM cases. If externally validated, this tool may have the potential value for creating larger cohorts of patients with GIM in an effort to better define the natural history of GIM and comparative outcomes based on practice patterns in the US. Finally, this study demonstrates the marked heterogeneity of biopsy techniques, pathology reporting, follow-up recommendations and HP treatment strategies following diagnosis of GIM, which likely is the effect of previous lack of formal accepted guidance. GIM is a fairly common incidental finding during EGD but is associated with a significantly increased risk of gastric cancer, particularly in patients with additional risk factors including corpus-extended GIM, family history of gastric cancer, persistent HP, among others. Until recently, the lack of established US guidelines left clinicians without clear guidance when confronted with this clinical scenario.

The prevalence of GIM diagnosis in this study was 2.5% of patients undergoing upper endoscopy during the study timeframe. While the prevalence of GIM in the U.S. population is unknown, this prevalence is lower than has been reported in previous U.S. cohorts, which has ranged from 5% to about 19% in various studies.[^9,24,25^](https://sciwheel.com/work/citation?ids=7944022,1954137,3554734&pre=&pre=&pre=&suf=&suf=&suf=&sa=0,0,0&dbf=0&dbf=0&dbf=0) Reported prevalence in international cohorts has varied even more widely, from 3.4% to over 30%[^9^](https://sciwheel.com/work/citation?ids=7944022&pre=&suf=&sa=0&dbf=0). These studies included patients undergoing EGDs for certain indications and had varying proportions of ethnic diversity included in their study populations compared to Utah which is ~85% non-Hispanic White. We also did not restrict our prevalence estimate to only those patients undergoing gastric biopsies, which presumably would bias towards a higher prevalence. Because this is a retrospective study, we could not control for the number or location of biopsies, which is relevant given that GIM is typically patchy and subject to sampling error.

As has been described in other studies, this study showed variability in recommendations from providers after diagnosis of GIM. A survey of gastroenterologists in the US in varying practice settings found confusion regarding the existence of guidelines, and variability in recommendations for surveillance.[^17,18^](https://sciwheel.com/work/citation?ids=6992304,3195680&pre=&pre=&suf=&suf=&sa=0,0&dbf=0&dbf=0) In a more recent retrospective study of patients with GIM, surveillance endoscopy was recommended in only about 17% of patients with intervals ranging from 1 to 4 years.[^26^](https://sciwheel.com/work/citation?ids=13296251&pre=&suf=&sa=0&dbf=0) A majority of patients (55%) in this study were not given clear guidance on when (or whether) to have an endoscopy repeated for surveillance. In addition, of ~ 1 in 4 patients who were recommended to have a surveillance endoscopy within a year, only about half completed this exam. This finding suggests that gastric cancer risk (such as country of origin, histology findings) is neither sufficiently characterized nor understood by providers who are making recommendations to patients. Additionally, it is unknown if patients are educated about their risk for developing gastric cancer. It is unclear what other factors endoscopists may have used in recommending surveillance endoscopies to some patients and not in others.

Endoscopy practice patterns, including biopsy sites and description of gross findings, along with histology practice patterns, including histologic subtyping of GIM and severity scoring, both vary widely. The risk of progression of GIM to gastric cancer has been demonstrated to be higher among patients with metaplastic changes in both the antrum and corpus.[^8,27^](https://sciwheel.com/work/citation?ids=1533622,7944023&pre=&pre=&suf=&suf=&sa=0,0&dbf=0&dbf=0) Despite this, the location of GIM was unspecified in over 40% of cases described. This can be explained by the fact that there has been shown to be a poor correlation between the endoscopic (visual) identification of GIM and a histologic diagnosis in the US particularly.[^28^](https://sciwheel.com/work/citation?ids=2969039&pre=&suf=&sa=0&dbf=0) Additionally, while histologic description of “limited” or “corpus-extended” GIM was used in 47% of cases, over half of the diagnoses were not specified; histologic subtype, complete vs incomplete, was not reported, even though this can be a marker of progression risk. While there are standardized biopsy approaches (updated Sydney protocol) and recommended quality performance measures for upper endoscopy, adherence to these is not evident in this study.[^29,30^](https://sciwheel.com/work/citation?ids=4958049,13296256&pre=&pre=&suf=&suf=&sa=0,0&dbf=0&dbf=0)

HP testing was clearly described in pathology reports in 86% of cases, and treatment was documented in almost 95% of patients; however eradication was confirmed in only 35%, owing to a very poor rate of performance for eradication confirmation testing. The European Society of Gastrointestinal Endoscopy (ESGE) strongly recommends to test for and eradicate HP once the diagnosis of GIM has been made.[^31^](https://sciwheel.com/work/citation?ids=6992307&pre=&suf=&sa=0&dbf=0) The recent AGA guidelines also strongly recommend HP treatment and confirmation of eradication based on high-quality evidence that treatment (regardless of GIM) is associated with reduced risk of gastric cancer.[^20^](https://sciwheel.com/work/citation?ids=7699366&pre=&suf=&sa=0&dbf=0) Whether or not histologic testing is sufficient in the setting of GIM to adequately assess for HP infection is unknown. In this study, there were no cases where a negative histologic examination for HP triggered further evaluation (ie via hydrogen breath test or stool antigen test).

The strengths of this study include the large cohort of patients diagnosed with GIM, which is among the largest in the US identified to date, and manual chart review to confirm the diagnosis and abstract patient-level data. The physicians involved in the study were all teaching faculty in a large University setting. An integrated EHR was available to obtain relevant patient and clinical data. The novel development of an NLP tool to meld endoscopy and pathology reports made identification of the study population achievable. One of the limitations of the study is its retrospective nature. As data were obtained from the EHR, and since the University of Utah serves a large catchment area, it is possible that not all follow-up data were captured. Although all endoscopies were performed using high-definition white light, the lack of standardization in EGD and biopsy practices (e.g. adherence to Sydney protocol, use of virtual chromoendoscopy, etc) and reporting of pathology specimens is also a limitation. However, we will note that the practice patterns we identified are unlikely to be unique to our practice setting and may even be more pronounced in some centers.

**Conclusion**

In conclusion, this study identifies significant unmet needs and points of intervention in the management of GIM. Notably, it highlights the lack of standardized care and follow-up recommendations preceding recently published US based guidelines. The study highlights the variability in clinical practice and future work should focus on understanding if the new US guidelines help providers and patients better understand GIM disease progression risk factors to allow for more standard follow-up recommendations. Additionally, clinicians should be educated about the need for documentation of HP testing and confirmed eradication of infection, particularly given the rising rate of HP eradication failure. Further studies are needed to determine and solidify appropriate recommendations for the management and surveillance of GIM.

Figure 1: Flow diagram of cohort selection

Table 1: Patient characteristics

Table 2: Endoscopy indications, findings and biopsy practice patterns

Table 3: GIM location and histology characteristics

Figure 2: Follow up recommendations

Table 4: *H. pylori* treatment outcomes

**References**

[1.    Sung, H. *et al.* Global cancer statistics 2020: GLOBOCAN estimates of incidence and mortality worldwide for 36 cancers in 185 countries. *CA Cancer J. Clin.* **71**, 209–249 (2021).](https://sciwheel.com/work/bibliography/10431948)

[2.    Stomach Cancer - Cancer Stat Facts. https://seer.cancer.gov/statfacts/html/stomach.html.](https://sciwheel.com/work/bibliography/6437304)

[3.    Lui, F. H., Tuan, B., Swenson, S. L. & Wong, R. J. Ethnic disparities in gastric cancer incidence and survival in the USA: an updated analysis of 1992-2009 SEER data. *Dig. Dis. Sci.* **59**, 3027–3034 (2014).](https://sciwheel.com/work/bibliography/2480997)

[4.    Shah, S. C. *et al.* Population-Based Analysis of Differences in Gastric Cancer Incidence Among Races and Ethnicities in Individuals Age 50 Years and Older. *Gastroenterology* **159**, 1705-1714.e2 (2020).](https://sciwheel.com/work/bibliography/9409962)

[5.    Pabla, B. S., Shah, S. C., Corral, J. E. & Morgan, D. R. Increased Incidence and Mortality of Gastric Cancer in Immigrant Populations from High to Low Regions of Incidence: A Systematic Review and Meta-Analysis. *Clin. Gastroenterol. Hepatol.* **18**, 347-359.e5 (2020).](https://sciwheel.com/work/bibliography/7530995)

[6.    Merchant, S. J. *et al.* A rising trend in the incidence of advanced gastric cancer in young Hispanic men. *Gastric Cancer* **20**, 226–234 (2017).](https://sciwheel.com/work/bibliography/4477347)

[7.    Anderson, W. F. *et al.* The Changing Face of Noncardia Gastric Cancer Incidence Among US Non-Hispanic Whites. *J Natl Cancer Inst* **110**, 608–615 (2018).](https://sciwheel.com/work/bibliography/4953358)

[8.    Gawron, A. J. *et al.* AGA Technical Review on Gastric Intestinal Metaplasia-Natural History and Clinical Outcomes. *Gastroenterology* **158**, 705-731.e5 (2020).](https://sciwheel.com/work/bibliography/7944023)

[9.    Altayar, O. *et al.* AGA Technical Review on Gastric Intestinal Metaplasia-Epidemiology and Risk Factors. *Gastroenterology* **158**, 732-744.e16 (2020).](https://sciwheel.com/work/bibliography/7944022)

[10.   Correa, P., Piazuelo, M. B. & Wilson, K. T. Pathology of gastric intestinal metaplasia: clinical implications. *Am. J. Gastroenterol.* **105**, 493–498 (2010).](https://sciwheel.com/work/bibliography/2280016)

[11.   Cho, E. *et al.* Cost-effectiveness outcomes of the national gastric cancer screening program in South Korea. *Asian Pac. J. Cancer Prev.* **14**, 2533–2540 (2013).](https://sciwheel.com/work/bibliography/2806399)

[12.   Zhang, X. *et al.* Endoscopic Screening in Asian Countries Is Associated With Reduced Gastric Cancer Mortality: A Meta-analysis and Systematic Review. *Gastroenterology* **155**, 347-354.e9 (2018).](https://sciwheel.com/work/bibliography/7531294)

[13.   Canakis, A., Pani, E., Saumoy, M. & Shah, S. C. Decision model analyses of upper endoscopy for gastric cancer screening and preneoplasia surveillance: a systematic review. *Therap. Adv. Gastroenterol.* **13**, 1756284820941662 (2020).](https://sciwheel.com/work/bibliography/9418815)

[14.   Shah, S. C., Canakis, A., Peek, R. M. & Saumoy, M. Endoscopy for gastric cancer screening is cost effective for asian americans in the united states. *Clin. Gastroenterol. Hepatol.* (2020) doi:10.1016/j.cgh.2020.07.031.](https://sciwheel.com/work/bibliography/9657736)

[15.   Saumoy, M. *et al.* Cost effectiveness of gastric cancer screening according to race and ethnicity. *Gastroenterology* **155**, 648–660 (2018).](https://sciwheel.com/work/bibliography/5401558)

[16.   Li, D. *et al.* Risks and Predictors of Gastric Adenocarcinoma in Patients with Gastric Intestinal Metaplasia and Dysplasia: A Population-Based Study. *Am. J. Gastroenterol.* **111**, 1104–1113 (2016).](https://sciwheel.com/work/bibliography/1481682)

[17.   Vance, R. B., Kubiliun, N. & Dunbar, K. B. How do we manage gastric intestinal metaplasia? A survey of clinical practice trends for gastrointestinal endoscopists in the united states. *Dig. Dis. Sci.* **61**, 1870–1878 (2016).](https://sciwheel.com/work/bibliography/6992304)

[18.   Shah, S. C., Itzkowitz, S. H. & Jandorf, L. Knowledge gaps among physicians caring for multiethnic populations at increased gastric cancer risk.](https://sciwheel.com/work/bibliography/3195680)

[19.   Tan, M. C., Mallepally, N., Liu, Y., El-Serag, H. B. & Thrift, A. P. Demographic and lifestyle risk factors for gastric intestinal metaplasia among US veterans. *Am. J. Gastroenterol.* **115**, 381–387 (2020).](https://sciwheel.com/work/bibliography/13296233)

[20.   Gupta, S. *et al.* AGA Institute Guidelines for Management of Gastric Intestinal Metaplasia. *Gastroenterology*.](https://sciwheel.com/work/bibliography/7699366)

[21.   Davitkov, P. *et al.* Advancing the Science in Gastric Pre-Neoplasia: Study Design Considerations. *Gastroenterology* **158**, 751–759 (2020).](https://sciwheel.com/work/bibliography/7969904)

[22.   von Elm, E. *et al.* The Strengthening the Reporting of Observational Studies in Epidemiology (STROBE) statement: guidelines for reporting observational studies. *Ann. Intern. Med.* **147**, 573–577 (2007).](https://sciwheel.com/work/bibliography/2432932)

[23.   Chapman, B. E., Lee, S., Kang, H. P. & Chapman, W. W. Document-level classification of CT pulmonary angiography reports based on an extension of the ConText algorithm. *J. Biomed. Inform.* **44**, 728–737 (2011).](https://sciwheel.com/work/bibliography/5653968)

[24.   Choi, C. E., Sonnenberg, A., Turner, K. & Genta, R. M. High prevalence of gastric preneoplastic lesions in east asians and hispanics in the USA. *Dig. Dis. Sci.* **60**, 2070–2076 (2015).](https://sciwheel.com/work/bibliography/1954137)

[25.   Fennerty, B. *et al.* Gastric Intestinal Metaplasia in Ethnic Groups in the Southwestern United States1.](https://sciwheel.com/work/bibliography/3554734)

[26.   Huang, R. J. *et al.* Prevalence, risk factors, and surveillance patterns for gastric intestinal metaplasia among patients undergoing upper endoscopy with biopsy. *Gastrointest. Endosc.* **91**, 70-77.e1 (2020).](https://sciwheel.com/work/bibliography/13296251)

[27.   Reddy, K. M., Chang, J. I., Shi, J. M. & Wu, B. U. Risk of gastric cancer among patients with intestinal metaplasia of the stomach in a US integrated health care system. *Clin. Gastroenterol. Hepatol.* **14**, 1420–1425 (2016).](https://sciwheel.com/work/bibliography/1533622)

[28.   Eshmuratov, A. *et al.* The correlation of endoscopic and histological diagnosis of gastric atrophy. *Dig. Dis. Sci.* **55**, 1364–1375 (2010).](https://sciwheel.com/work/bibliography/2969039)

[29.   Rugge, M. *et al.* Operative link for gastritis assessment vs operative link on intestinal metaplasia assessment. *World J. Gastroenterol.* **17**, 4596–4601 (2011).](https://sciwheel.com/work/bibliography/4958049)

[30.   Bisschops, R. *et al.* Performance measures for upper gastrointestinal endoscopy: A European Society of Gastrointestinal Endoscopy quality improvement initiative. *United European Gastroenterol. J.* **4**, 629–656 (2016).](https://sciwheel.com/work/bibliography/13296256)

[31.   Pimentel-Nunes, P. *et al.* Management of epithelial precancerous conditions and lesions in the stomach (MAPS II): European Society of Gastrointestinal Endoscopy (ESGE), European Helicobacter and Microbiota Study Group (EHMSG), European Society of Pathology (ESP), and Sociedade Portuguesa de Endoscopia Digestiva (SPED) guideline update 2019. *Endoscopy* **51**, 365–388 (2019).](https://sciwheel.com/work/bibliography/6992307)

**Declarations**

**Ethics approval and consent to participate**: This retrospective cohort study was approved by the University of Utah Health Institutional Review Board

**Consent for publication**: All listed authors consent to publication.

**Acknowledgements**: None

**Funding**: SCS has effort supported by a Veterans Affairs Career Development Award (ICX002027A) and an American Gastroenterological Association Research Scholar Award.

**Competing Interests/Disclosures**: SKP: none to disclose; MJS: none to disclose; JCF: Advisor for Obalon Therapeutics and Veritract Inc.; KAP: none to disclose; SCS: Ad hoc consultant to Phathom Pharmaceuticals and RedHill BioPharma; AJG: none to disclose

**Availability of data and material**: data are not publicly available due to protected health information. The corresponding author can be contacted if additional materials and methods information is needed.
